# Medication administration in nursing homes: A qualitative study of the nurse role

**DOI:** 10.1002/nop2.216

**Published:** 2018-11-28

**Authors:** Kristian Ringsby Odberg, Britt Sætre Hansen, Sigrid Wangensteen

**Affiliations:** ^1^ Department of Health Sciences Norwegian University of Science and Technology (NTNU) Gjøvik Norway; ^2^ Faculty of Health sciences, SHARE—Centre for Resilience in Healthcare University of Stavanger Stavanger Norway

**Keywords:** medication, nurses, nursing, nursing homes, older people

## Abstract

**Aims:**

The objective of this study was to expand the knowledge of the nurse role during medication administration in the context of nursing homes. The following research question guided the study: *How can the nurse role during medication administration in nursing homes be described?*

**Design:**

A QUAL–qual mixed study design was applied.

**Methods:**

Data were collected using partial participant observations and semi‐structured interviews of all staff members involved in medication administration. An inductive content analysis was performed.

**Results:**

Medication administration is a pervasive process ingrained in the day‐to‐day activities of providing care to the patients. The nurse role is compensating, flexible and adaptable. There is a dynamic interaction between several contributory factors, those being shifting responsibility, a need for competence, invisible leadership, varying available competence, staff stability and vulnerable shifts.

## 1 INTRODUCTION

Patient safety issues in primary health care are mainly related to diagnosis and medication. It is generally acknowledged that adverse events related to medication administration account for a significant threat to overall patient safety (Kohn, Corrigan, & Donaldson, 2000; Makeham, Dovey, Runciman, & Larizgoitia, 2008; Marchon & Mendes, 2014; Vogelsmeier, 2014). Medication administration involves an intricate mixture of various tasks and demands that temporally structure the nurse’s workday (Carayon et al., 2014; Grigg, Garrett, & Craig, 2011; Jennings, Sandelowski, & Mark, 2011; Moyen, Camiré, & Stelfox, 2008; Odberg, Sætre Hansen, Aase, & Wangensteen, 2017).

Primary health care in the Western World reaches out to a broad segment of the population and is the facet of the healthcare system with which most people interface. Each municipality independently governs Norwegian nursing homes, and there are local and regional variations in size, patient types and the style of management. However, the basic principles of active treatment and ensuring the basic needs of the residents are universal (Malmedal, 2014). Recent reforms have led to increased collaboration between primary care and specialist health care. Nursing homes experience increased pressure to receive more patients needing more complex active medical treatment, compared with a few years back (Syse & Gautun, 2013).

## 2 BACKGROUND

The medication administration process consists of six stages: ordering and prescription; transcribing; dispensing; preparing; administering; and finally observing and documenting effects and side effects (Carayon et al., 2014). Medication administration errors (MAE) may occur anywhere along this chain and cause an adverse drug event (ADE; Carayon et al., 2014; Choo, Hutchinson, & Bucknall, 2010; Odberg et al., 2017; Smeulers, Onderwater, Zwieten, & Vermeulen, 2014). According to WHO (2016), MAE’s are preventable at different levels.

Overall research acknowledges the importance of the nurse role in maintaining and improving medication safety in health care (Choo et al., 2010; Grigg et al., 2011; Kowalski & Anthony, 2017; Smeulers et al., 2014). Many factors influence safe medication management. Some argue that nurses (RN) may have insufficient knowledge and skills to perform safe medication management (Andersson, Frank, Willman, Sandman, & Hansebo, 2018; Simonsen, 2016); others point to normalization of risk‐inducing behaviour and interruptions (Odberg et al., 2017), or use of technology, design flaws, time constraints, poor communication, lack of leadership, as well as outdated policies and guidelines (Al‐Jumaili & Doucette, 2017; Carayon et al., 2014; Keers, Williams, Cooke, & Ashcroft, 2013; Lapkin, Levett‐Jones, Chenoweth, & Johnson, 2016; Marasinghe, 2015). There is an apparent lack of studies investigating the nurse role during medication administration in nursing homes.

Due to the complexity of medication administration, the acknowledgement of MAE’s in primary care and the essential role of the RN, the objective of this study was to expand knowledge of the nurse role during medication administration in the context of nursing homes. The following research question guided the study: How can the nurse role during medication administration in nursing homes be described?

## 3 METHOD

### 3.1 Design

The study applied a qual‐qual mixed method design (Morse, 2016) using partly participant observations (Hammersley & Atkinson, 2007) supplemented by semi‐structured interviews for data collection. The first author collected all the data in two nursing home wards in Eastern Norway.

### 3.2 Study setting and recruitment

The senior managers of the participating nursing homes were contacted by telephone in December 2015. They were informed of the objective and content of the study and agreed to participate. Shortly after, the first author briefed the entire staff on both wards during regular staff meetings and asked whether they would consider participating in interviews. One nursing home ward with ten patients was rurally based and catered mostly to patients suffering from dementia and minor disabilities. The other nursing home ward, with six patients, was in a neighbouring urban municipality, with patients having multiple complex medical diagnoses and in need of palliative care.

### 3.3 Data collection methods

A pilot study was conducted in a nursing home ward providing a similar contextual setting as the current study to test the data collection methods. Experiences and findings from the pilot study resulted in a more detailed observation guide and interview guide. No data from the pilot study were used in the current study.

The data collection took place in 2016, consisting of 140 hr of observations supplemented by 16 semi‐structured interviews of staff members. Most observations took place in the daytime shift and a few on the evening shift and opening hours of the night shift. The first author, dressed in work attire, followed staff members around conducting partly participating observations during medication administration‐related tasks (Hammersley & Atkinson, 2007). A semi‐structured observation guide based on the elements in the work system of Human Factors theory (persons, tasks, physical environment, tools and technology, organization) guided the researcher when observing the different stages of medication administration (Carayon et al., 2006). Examples are, observations of pre‐visitation, transcribing medicines or staff preparing medicines before administering them. Situations observed were noted between sessions, while excerpts from relevant conversations between staff members were written down verbatim immediately. After each observational session, all notes were transcribed and expanded on while the memory of the events was clear in the mind.

Participants working more than a 50% position for more than a year were interviewed. There were eight staff nurses, three nurse assistants, two nurse managers and two doctors. The majority were women (12). The reason for including professions apart from the nurses was observations showing a strong dynamic interaction between all staff members during medication administration. The interviews were digitally recorded and lasted from 30 min ‐ 1 hr. The interview guide was constructed in line with observational findings and from elements in the work system in Human Factors theory (Carayon et al., 2006).

### 3.4 Analysis

Shortly after finalizing the data collection, the authors read all the material multiple times to reach a common understanding of the data as a whole. The first author then coded openly in the margins of the transcribed material, extracting meaning units pertaining to the research question. These meaning units were condensed, coded and grouped based on similarities, forming subcategories and main categories in line with principles in inductive content analysis (Elo & Kyngäs, 2008). Data from the observations and interviews were handled and coded separately and integrated in the final stage of the categorization process (Morse, 2016). Analytical discussions and reflections with the co‐authors led to several iterations before arriving at a conceptual model. Observational data formed the core for describing the day‐to‐day care and the structure of medication administration. Excerpts from the interviews and observation notes were chosen to illustrate the different main categories and subcategories. They are reported in italics throughout the Results section and coded to differentiate the position (second and third letter) and the individuals (final letter):

IRN‐A = Interview Registered Nurse A
INA‐A = Interview Nurse Assistant A
INM‐A = Interview Nurse Manager A
IMD‐A = Interview Medical Doctor A

An example of analysis is shown in Table 1.

**Table 1:** Analysis exemplified with one of three main categories and subsequent subcategories

Main category	Sub‐category	Condensed meaning	Examples of meaning units
Compensating	Need for competence	Differences in individual competencies. Keeping up to date is an individual responsibility	IRN‐D Yeah…internal education, we have some of that. The previous doctor used to spend some time with us, refreshing competencies and skill—not anymore though—and sometimes we arrange some educational stints
	Shifting responsibility	The nurse is regarded as pivotal for the running of day‐to‐day business	IRN‐E It may be slow at times if the doctor is uncertain. He does not take hasty or quick decisions and may sow doubt by the way he acts. Then you feel more responsible as a nurse, because you have to lead the way somehow, and that is not how it should be

### 3.5 Ethics

The Norwegian Social Science Data Service (NSD; No. 45389) approved the study. Since there was no involvement of patients or use of patient information, the study did not require approval from the Norwegian Regional Committee for Medical Health Research Ethics.

The first author is a male registered intensive care nurse with no prior familiarity with or knowledge of any of the wards or the participants in the study. All participants gave their informed consent and were informed of data confidentiality and of the opportunity to withdraw at any time. No one chose to withdraw during or after data collection.

Before observations, the researcher informed all participants that professional ethics overrode researcher neutrality, meaning that the staff would be alerted if the researcher identified situations where patient harm could be averted (Guillemin & Gillam, 2004). The researcher encountered no such situations.

The paper was prepared according to SRQR guidelines (O’Brien, Harris, Beckman, Reed, & Cook, 2014).

## 4 RESULTS

When aiming to describe the nurse role in medication administration, three main categories emerged: compensating, flexible and adaptable. Each of these main categories contains subcategories describing different aspects of the nurse role and the collaboration needed to perform medication administration. The results reflect a dynamic interaction of several contributory factors and how the nurse role is integral in medication administration as shown in Table 2:

**Table 2:** Contributory factors influencing the nurse role during medication administration on different levels

Individual level **Compensating** Need for competence Shifting responsibility	Team level **Flexible** Leadership Available competence	Organizational level **Adaptable** Staff stability The vulnerable shifts
Varying competence Need for updated competence Medication administration perceived as complex by RN’s Takes on more responsibility than necessary Administrative tasks take precedence The RN’s are natural leaders Do more tasks than obliged Inadequate resources	Leadership is distributed and invisible Nurse managers are in a tight position Delegation of tasks Available competence Vulnerable Random Informal leadership Random team composition RN’s prioritize administrative tasks	Shifting workload Cannot plan for everything Staff stability important Experience and personality Staff composition important Workarounds are normal Prepare in advance Contingency plans Continuity of care

### 4.1 Compensating

The roles of the individual staff members are affected by the competencies of the surrounding staff. The most striking finding is how the nurse in charge is left to compensate for the degree of skills and competencies of their team members.

#### 4.1.1 Shifting responsibility

NA’s perceive medication administration as an easy task, describing it as only preparing and administering medicines. The nurses have a fuller picture encompassing all six stages of the medication administration process, and they also consider it a much more complex process as documented in the following interview excerpts with a nurse:

IRN‐A I started out as an NA, which I appreciate. It gave me a lot of the basic skills necessary, but of course, there is a lot more responsibility as a nurse. You do more of the same, but you have more responsibility and more tasks as a nurse.

The NA’s see themselves in the light of the nurses and perceive their duty to assist the nurses. Consequently, they consider the nurses to be their superior in all settings, referring to them if questions or problems arise. Some nurses thrive on this, making them feel competent and taking the role as leaders. This invisible role designation led to a hierarchical structure, especially evident on shifts with a single nurse. On shifts with several nurses, seniority seems to fall to the nurse with most experience as illustrated in this observational excerpt:

There are three nurses in the nurse station, allocating tasks at the start of the morning shift. It is hard to identify who is the leader, but after a while, the nurse with seniority becomes the centre of attention and makes final decisions on which patients they will have responsibility for.

The nurses have a considerable responsibility, and they tend to take on tasks belonging to the other staff members as well as their own. Observations document that the nurses often regard themselves as being “the spoke of the wheel” and often define specific medication administration tasks as more important than other tasks. A substantial number of the tasks related to medication administration were delegated from the MD and could not be delegated to nurse assistants.

The nurses adjust dosages to patients with varying needs, for example, when administering drugs for diabetes or pain management. Most often, they have a sheet of paper with pre‐authorization from the doctor on various drugs. At other times, the nurses make changes or adjustments themselves, based on observations and patient needs and inform the doctor on a later occasion. Excerpt from observational notes:

During pre‐visitation the nurse informs the doctor that “we have made the following changes in some medication prescriptions. The nurse then asks the doctor if he may formalise the changes, which means to transcribe them in the electronic medication administration record. Then the nurse rationalises the decision and the doctor agrees.

The MD generally accepts this as normal routine provided the RN’s are able to substantiate the drug alterations. An excerpt from an interview with an MD follows:

IMD‐A I know how experienced the nurses on this ward are when it comes to administering morphine, so I probably often note the indication and give the nurses space to be flexible. There is seldom a right or wrong, but the nurses have to substantiate their opinions or when they make alterations.

Observations documented that when the doctor was uncertain, the nurses experienced more responsibility together with a feeling of uneasiness. In cases where the doctor had strong opinions and openly discussed the patients with the nurses, they were included and empowered. This duality gave rise to the nurses compensating for how the doctor behaved. If they considered the doctor to be “weak,” they compensated by taking on tasks that were not theirs initially. If they considered the doctor “strong,” they let the doctor handle things as they stood. Examples of additional tasks could be how the nurse offered to take on documentation tasks belonging to the doctor (transcribing), merely to ensure that this was done.

#### 4.1.2 Need for competence

The staff often noted that patients have more diagnoses and are in need of more advanced medication administration than before; they had to take responsibility for patients before they were adequately treated or diagnosed and in turn more complex tasks related to medication administration. This has led to more responsibility and a need for updated competence.

There is limited funding to send staff to courses and conferences and maintaining competence largely depends on personal initiative. The staff complain that if they need more advanced competence, they have to use their spare time, receiving no financial reimbursements or incentives. At the same time, all staff members acknowledge that complex healthcare environments and nursing sciences are in constant flux due to advances both medically and procedurally.

The managers seemed aware of the inadequate resources that inhibit competence development in the staff, placing them between a rock and a hard place. One nurse manager described it in an interview as:

INM‐A We continuously receive new guidelines relating to medications, with new demands on documentation. At the same time, we need to keep tabs on everything; it always comes down to the economy, who pays for what. Everything has consequences if we are not thorough in following up. We have more tasks and demands than ever.

### 4.2 Flexible

Flexibility mirrors the freedom staff members experience in structuring their workday and performing medication‐related activities. Tasks in the workgroup on specific shifts are delegated differently in line with changing circumstances. The nurse also compensates for the other team members’ strengths and weaknesses. If a nurse spots a weakness in a colleague or does not trust him or her to do a specific task, they do it themselves instead. When they did, it was not explicitly stated and was viewed by the others as expected behaviour.

#### 4.2.1 Available competence

The team on a specific shift have a shared world of experience and skill where the staff works. Available skills and competencies on a given shift are demarcated partly by the professions in the team.

Some shifts may experience staff lacking the competencies to administer certain medications. At other times, only one person, usually a nurse, has the necessary skills to perform specific activities vital to a patient. This may lead to vulnerability as the team may experience a lack of skill redundancy. Such vulnerability may lead to adverse events under adverse circumstances, for example, staff shortage, or unexpected events in the ward. Some shifts have only one nurse, and most administrative and medication‐related tasks will fall on that nurse. Many tasks during a shift are indirectly care‐related or related to medication administration; these are perceived as administrative tasks. Administrative tasks are often considered a nurse prerogative, and nurses may find themselves swamped because of their inherent task flexibility, being able to undertake a variety of roles. If there are NA’s present, they are most often engaged in clinical work, close to the patient, reporting verbally to the nurse on the team. The NA’s acknowledge the nurses’ workload:

INA‐A If you have the evening shift alongside a nurse, they have a higher workload, because a majority of the activity on this ward demands a nurse, because of competence and such.

#### 4.2.2 Leadership

The nurse managers were in charge of the team composition on the individual shifts, distributing staff across the various shifts, weeks in advance. The teams were formed so that professions complemented each other with the aim of always having a nurse on all shifts.

Although the staff are supposed to update on the patients on their own by reading from the electronic medical record, they also had an informal roundtable discussion before commencing each shift. This discussion served to vent frustration, to reflect on recent events, but also to discuss and delegate patients and specific tasks among the staff members. The task‐allocation often took into account the wishes of the staff members and was in contrast to the manager’s prior assignments:

INA‐A “Patients and tasks are in fact assigned in advance, but we sit there during the time of the report and distribute tasks and patients among ourselves as well. It depends on the workload, if our wishes are granted, we have to ensure that no one gets too much to do, that we assign fairly. If we have a nurse on that shift, she will have the final say. Otherwise, it's like the toss of the dice.”

The skills and competencies available on a particular shift result from the managers’ pre‐planning but get randomized as circumstances change; staff may become ill, forcing changes. The flexibility of task assignment is therefore dependent on the skills and competencies needed in the various tasks related to medication administration. Not all staff members can set up an intravenous line or administer all type of medicines.

### 4.3 Adaptable

The main category “adaptable” contains two related categories: *Staff stability* and *Vulnerable shifts*. In short, adaptability is about how the staff adapt to changing workloads during the various shifts and how they perceive the relationship with their co‐workers as a critical factor in collaborating and performing medication administration safely. An alteration in work tasks and workload is sometimes predictable, but most often not. Consequently, some shifts end up being vulnerable.

#### 4.3.1 Staff stability

Staff stability is critical to achieving optimal care for the patients, underlining the importance of knowing your co‐workers when working in a demanding and complex environment. Working well together depends on personality, and there are individual differences influencing cooperation. The freedom to ask colleagues for help during medication administration is reported as crucial by most staff members and depends on a shared understanding of the situation and that all staff members report on their location at all times. Also, sharing experiences together seems vital, allowing the staff to form bonds that would not otherwise have formed. The relationship with co‐workers is illustrated in the following excerpt from an interview with a nurse assistant:

INA‐B “We experience a lot together, stressful and taxing situations…for the most part we are good at talking to each other, but there are variations, it depends on who you're working with; it's all about personal chemistry.”

Having good personal chemistry with colleagues was necessary for the staff to thrive. When the staff know each other, they are less vulnerable if something unpredictable happens. The quality of the care depends on the stability of the staff and when staff members know each other, there seems to be less need for direct communication and delegation of tasks. A stable staff also know the patients and can work more efficiently and may provide better care. The opposite happens if there are many substitute nurses; the continuity of care may be disrupted and a proportionally higher fraction of the total workload is taken on by the regular staff members.

#### 4.3.2 The vulnerable shifts

In periods of high workload, the staff seems to work with great efficiency and they describe the work as going smoothly. Like one nurse said: *IRN‐B* “When it’s busy we are like well‐oiled machinery.” Another nurse stated that it is a balancing act. “If it’s too hectic, we do not work so well together”. Such high workloads may have positive professional outcomes, as the staff claim to work more smoothly. It may also lead to adverse patient outcomes in that the healthiest patients receive less attention and care. One nurse (IRN‐C) said during observations that *“*when it is busy we prioritise medication to the patients most needing it.” At the same time, several stated that they like working when it is busy since it gives them a feeling of higher self‐worth.

Both nursing home wards reported staff levels to be adequate during the day shifts on weekdays. Evening shifts, night shifts and weekends were often reported as vulnerable depending on workload and status of the current patients. This vulnerability was directly linked to the professions and competencies of the staff at work. Working vulnerable shifts seemed to invoke negative emotions in the staff and an excerpt from an interview with a nurse describes it as follows:

IRN‐D “This is the way it is. I feel very alone during my weekend shifts, being a single nurse and the only regular staff member. That is not okay. I feel that I lose control and when Monday finally arrives, I send a silent thanks that everything went well.”.

Some night shifts had no nurse on duty, and all medications had to be prepared in advance. The staff were aware of the vulnerable shifts in advance and did their best to plan accordingly, as shown in this observation note:

The nurse in charge realises that there are no nurse set up on the next shift and that they have a patient suffering from pains hard to relieve. They decide to prepare a dose of morphine in advance, doing the double‐checking now.

This proactive engagement seems to be due partly to the unpredictable nature of working in a complex healthcare system; the staff expected the unexpected.

Because the vulnerable shifts could be particularly unpredictable, the staff prepared medications in advance or sent notice to the staff on the neighbouring wards that they might need assistance. In coping with the provision of medicines around the clock, the staff knowingly bent guidelines and procedures to fit the reality of their work environment. An excerpt from an interview with a nurse elaborates on how she would handle a potential situation on a vulnerable shift:

IRN‐E If I needed to administer morphine and was alone on my shift, I might have taken a photo with my cell phone and sent it to a colleague for confirmation. I would have done something like that if the situation demanded it.

## 5 DISCUSSION

The main findings indicate that the RN has a central role at all the stages of medication administration and that this role goes beyond the job description. Varying workload, staff stability, the degree of leadership, available competence and dynamic events in the workday are compensated by the RN’s to ensure fulfilment of all tasks related to medication administration at all times.

### 5.1 Resilience

Medication administration in nursing homes is a complex process taking place in a complex system with inherent vulnerabilities, placing high demands on the sociotechnical work system and the staff (Carayon et al., 2014; Choo et al., 2010; Grigg et al., 2011; Odberg et al., 2017). Findings in the current study document this complexity and elaborate on how the staff and particularly the RN’s adjust to shifting circumstances in their work environment. Human Factors focus on the interaction of the elements in the sociotechnical work system and how people perform processes in this system (Carayon et al., 2006). Workarounds and adaptations are often described as “filling in the gaps” to cover for design flaws or internal or external pressure and complexity (Rankin, Lundberg, Woltjer, Rollenhagen, & Hollnagel, 2014). The main categories in the current study describe role compensation, flexibility and adaptability as crucial when describing the nurse role in medication administration. These categories reflect an intrinsic ability to confront and adjust to a dynamic and challenging workday.

If one adopts a resilience engineering perspective, work processes in complex systems are recognized by variations, driving people to change and adapt behaviour to meet the fluctuations both long‐term and short‐term (Hoffman & Woods, 2011). Everyday adaptations to cope with dynamic events can be described as performance variability, encompassing individual adaptations and how the surroundings react to them (Hollnagel, 2009, 2014 ). The nurse role is highly regulated, but the unpredictable nature of healthcare systems often forces RN’s to improvise, to find workarounds and adapts to the conditions offered by the current situation (Lindblad, Flink, & Ekstedt, 2017). Sometimes these adaptations may lead to unsafe situations, but most often they will have a successful outcome (Hollnagel, 2009).

**Figure 1:**
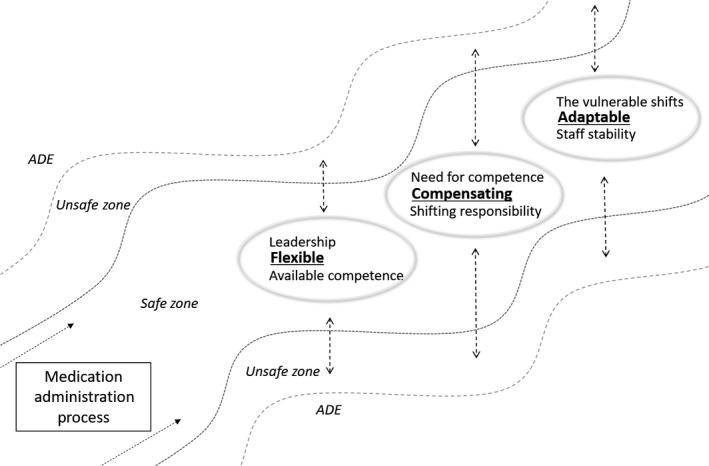
The balancing act of safe medication administration

Performance variability in a system should aim to be proportional to the system complexity, meaning that the staff of the nursing homes should have appropriate skills, resources and flexibility at hand to meet any unforeseen events (Braithwaite, Wears, & Hollnagel, 2016; Grigg et al., 2011). The current study identified six areas (subcategories) necessitating adaptive behaviour to ensure safe medication administration. These areas are on an individual level (Need for Competence and Shifting Responsibility), team level (Leadership and Available Competence) and organizational level (Staff Stability and The Vulnerable Shifts). Figure 1 illustrates the balancing act of safe medication administration documented in the study.

### 5.2 The nurses are compensating

Individual adaptive behaviour manifested itself in the degree of flexibility nurses exhibited about the medication administration responsibility and how they compensated for the other staff members. This flexibility depended on the capabilities of the workgroup on a specific shift, as well as their training and competence. Other attributes usually associated with nurses’ performance are motivation, fatigue and stress (Al‐Jumaili & Doucette, 2017; Carayon et al., 2006; Grigg et al., 2011). Furthermore, the training and skill maintenance in medication administration‐related tasks are to some degree random in that it is voluntary to participate. Consequently, the staff members may have different skill sets and competencies. Over time, this may contribute to lowering the overall competence of the staff.

Individual characteristics of the staff, therefore, vary significantly from shift to shift, having a impact on performance variability and degrading the ability to prepare for unexpected conditions. Changing circumstances meant that the staff had to improvise and prioritize. At the same time, the staff were obliged to undertake a variety of tasks, not all of them clinically related. These findings seem universal as RN’s often are required to undertake multiple tasks simultaneously in stress‐inducing physical environments, making them more prone to making errors (Carayon et al., 2014; Monroe & Graham, 2005; Odberg et al., 2017). Under high workload, administrative tasks related to medication administration took precedence for the RN’s, thus delegating the remaining workload to the other staff members. In effect, administration of drugs and the subsequent observations were delegated to RN’s or NA’s without first‐hand knowledge of the patients. A lack of task redundancy often resulted in task vulnerability, and medications or treatments sometimes had to be postponed or were interrupted. Breaks in the medication administration chain may increase the risk of committing MAE’s and potential ADE’s (Carayon et al., 2014).

### 5.3 The nurses are flexible

An important finding was how the leadership was distributed and invisible, leading to flexibility when delegating tasks and responsibilities. Nurse managers had indirect control of staff allocation and task delegation in that the staff often made their own decisions and planned contrary to prior assignments. The leadership and style of management seem to affect how the staff perform and delegate tasks. A clear leader with a hands‐on approach may impose more direct control and strictures in relation to the myriad of regulations and guidelines on medication administration, while a more distant leader lets the staff regulate more independently. In terms of resilience, this resembles the terms work‐as‐done (WAD) and work‐as‐imagined (WAI; Braithwaite et al., 2016). Human Factors theory often uses the analogues “blunt end” and “sharp end” to encapsulate much of the same meaning (Rankin et al., 2014; Reason, 2000). In the current study, the nurse managers of both nursing homes “imagined” how the wards should be run (WAI), something that not always translated to how it was actually done (WAD). This discrepancy underlines the importance of communication across levels and management capable of addressing the needs of the staff (Backman, Sjögren, Lövheim, & Edvardsson, 2017; Hollnagel, 2012). Examples in the current study indicate that even though managers endeavour to structure the workday of the staff, they simultaneously encourage flexible behaviour without giving clear indications of where this delineation ought to be. The staff may perceive this as distant management and thus use considerable internal resources to structure their workday. This entails the staff forming ad hoc teams with a random team‐structure and performing many of the tasks of the regular nurse manager.

### 5.4 The nurses are adaptable

The vulnerable shifts are to some degree predictable, but still pose challenges to the staff. Staff shortage, lack of competence and scarce resources may impede the staff’s ability to be adaptive and find workarounds (Hollnagel, 2009). Over time, this behaviour may evolve to be a part of normal operations, stretching the boundaries of safe medication administration. As a consequence, the staff may be balancing precariously close to unsafe medication administration in their daily routines without knowing. If something unpredictable happens during a vulnerable shift, the border may be crossed and ADE’s occur. Some staff members expressed gratitude when they finished a so‐called vulnerable shift and opined that sometimes it was due to luck or coincidence that no ADE’s occurred.

Staff stability and shared mental models are often recognized as a key factor to ensure safe care in healthcare environments (Salas & Frush, 2013). When the staff know each other’s skills and competencies and trust each other, there is less need for communication to coordinate medication administration tasks. They describe it as working in silent agreement. It may lead to increased freedom and flexibility when performing tasks, but may also lead to less structure, less use of guidelines, checks and regulations. The law of requisite variety states that WAI should be as complex or varied as WAD, meaning that one should strive to increase the knowledge and competence of the staff to enable them to cope with unforeseen activities. Another approach is to seek to minimize unforeseen events through rules, regulations, standardizations and guidelines (Braithwaite et al., 2016). To balance the complexity of the WAD and WAI, one needs an in‐depth understanding of the organization. Without it, medication administration may spiral into an unregulated activity, having both positive and negative effects—the positive effects being apparently increased resilience when facing unexpected events, the negative effects being the erasing of borders between safe and unsafe acts. Erasing the borders may continue and eventually breach the bounds of safe medication administration without the staff knowing. This may be exemplified by the RN who in a potential situation would consider using the mobile phone to message an image to a colleague rather than asking the manager to double‐check a medication to be a reasonable solution.

## 6 LIMITATIONS

Data collection was performed by a single researcher with a nursing background, which may introduce bias. This was countered by a research team, discussing and reflecting on the data throughout the research process. Having a nursing background may influence preconceptions, but also allows for rapidly gaining insights that might otherwise be missed. The researcher was aware of the potential Hawthorne effect throughout the observations. The two nursing home wards included were intentionally different, to provide a broad picture of the nurse role in medication management.

## 7 CONCLUSION

Medication administration is ingrained in normal clinical activities, and isolated work processes may be challenging to define. Work system factors such as competence, leadership and staffing may influence the ability to perform safe medication administration. To counter this, nurses exhibit role compensation and flexibility and are highly adaptable during all the stages of administering medicines. The seeming resilience nurses exhibit, may be brittleness, extending the boundaries of day‐to‐day clinical activities close to the borders of safe medication administration.

By identifying normal operations, one may learn, adapt and develop appropriate safety measures in the future. The study underscores the importance of first‐hand knowledge of the clinical setting before implementing interventions or enforcing any organizational changes.

## CONFLICT OF INTEREST

There are no conflict of interests.
